# Severe hypercalcemia as the initial presentation of renal cell carcinoma: a diagnostic case report

**DOI:** 10.1097/MS9.0000000000003252

**Published:** 2025-04-04

**Authors:** Bal Krishna Subedi, Shivani Modi, Naveen Gautam, Anuja Upadhyay, Paul Baek, Daniel Bitetto

**Affiliations:** a Department of Internal Medicine, Jefferson Einstein Montgomery Hospital (Einstein Medical Center Montgomery), East Norriton, Pennsylvania, USA; b Medical Officer, Gulmi Durbar Basic Hospital, Gulmi, Nepal; c ENT, Tribhuvan University, Institute of Medicine, Kathmandu, Nepal

**Keywords:** hypercalcemia, metastatic RCC, paraneoplastic syndrome, pTHrP, renal cell carcinoma

## Abstract

**Introduction and importance:**

Renal cell carcinoma (RCC) represents 90% of renal malignancies with rising global incidence. While the classic triad includes hematuria, flank pain, and palpable masses, paraneoplastic hypercalcemia occurs in 17% of cases and indicates aggressive disease behavior. This case documents severe hypercalcemia as the initial manifestation of metastatic RCC.

**Case presentation:**

A 64-year-old male presented with malaise, bloating, and weight loss. Laboratory evaluation revealed severe hypercalcemia (15.1 mg/dL), suppressed parathyroid hormone (4.2 pg/mL), and elevated parathyroid hormone-related peptide (83 pg/mL). Imaging identified a 5.5 × 5.0 × 5.3 cm left renal mass with metastases to lungs, brain, and possibly bone. Biopsy confirmed metastatic clear-cell RCC. Management comprised aggressive hydration, zoledronic acid, combined immunotherapy (pembrolizumab/lenvatinib), and palliative radiation for cerebral lesions.

**Clinical discussion:**

This case illustrates PTHrP-mediated humoral hypercalcemia of malignancy in metastatic RCC. The pathophysiology differs from alternative mechanisms such as calcitriol-mediated hypercalcemia or cytokine-driven osteoclast activation. Diagnostic markers demonstrated a classic humoral pattern with PTHrP elevation (83 pg/mL) and PTH suppression (4.2 pg/mL). The multimodal imaging protocol effectively delineated primary tumor dimensions, vascular invasion, and metastatic burden. Treatment efficacy was objectively measured through serial calcium levels and documented radiographic regression of primary and metastatic lesions. This aligns with recent data on combination immunotherapy (anti-PD-1) with tyrosine kinase inhibition in advanced RCC, supporting current therapeutic paradigms for PTHrP-mediated hypercalcemia in metastatic disease.

**Conclusion:**

Prompt recognition of paraneoplastic hypercalcemia in RCC facilitates timely intervention. This case demonstrates the value of comprehensive diagnostic evaluation and multidisciplinary management combining supportive care with targeted immunotherapy. Further prospective studies are needed to optimize therapeutic strategies for patients presenting with metabolic derangements suggestive of underlying malignancy.

## Introduction

Renal cell carcinoma (RCC) constitutes over 90 percent of all renal malignancies^[1]^ and accounts for approximately 3% of all cancers worldwide^[2]^. Its incidence is rising globally, with a reported annual increase of 400 000 new cases and a worldwide mortality rate approaching 175 000 deaths per year^[3]^. RCC predominantly affects individuals aged 55–74 years, with a male-to-female ratio of roughly 2:1^[4]^. Key risk factors include cigarette smoking, obesity, hypertension, occupational exposures such as trichloroethylene, and inherited syndromes such as von Hippel-Lindau disease and tuberous sclerosis^[1]^.

**HIGHLIGHTS**

- **Rare presentation**: A 64-year-old male presented with severe hypercalcemia (serum calcium >15 mg/dL) as the initial manifestation of metastatic clear cell renal cell carcinoma (RCC).
- **Diagnostic challenge**: The absence of classic RCC symptoms (hematuria, flank pain, palpable mass) posed a diagnostic challenge.
- **Paraneoplastic hypercalcemia**: Elevated parathyroid hormone-related peptide (PTHrP) levels confirmed the paraneoplastic nature of the hypercalcemia.
- **Extensive disease**: Imaging revealed pulmonary nodules, renal vein thrombosis, adrenal involvement, and brain metastases.
- **Multidisciplinary management**: Successful treatment involved a combination of immunotherapy (pembrolizumab), targeted therapy (lenvatinib), and supportive care including zoledronic acid for hypercalcemia.
- **Clinical implications**: This case underscores the importance of considering malignancy in patients with unexplained hypercalcemia and highlights the need for a comprehensive diagnostic approach and multidisciplinary management in advanced RCC with paraneoplastic syndromes.

While the classic triad of hematuria, flank pain, and palpable masses is well recognized, only 10–15% of patients present with these symptoms at diagnosis^[1]^. Notably, paraneoplastic manifestations such as hypercalcemia are observed in approximately 17% of cases^[5]^. Severe hypercalcemia with serum calcium levels often exceeding 14 mg/dL is primarily mediated by the overproduction of parathyroid hormone-related peptide (PTHrP), compounded in some instances by cytokine-induced osteoclast activation^[5,6]^.

Diagnostic evaluation relies on high-resolution imaging modalities, including contrast-enhanced computed tomography (CT) and magnetic resonance imaging (MRI), to delineate the tumor extent and metastatic spread^[1]^. Biopsy remains essential for histopathological confirmation, particularly in cases with atypical presentations or when metastases are evident. Serum biomarkers, such as PTH and PTHrP, further aid in differentiating malignancy-associated hypercalcemia from primary hyperparathyroidism^[6]^.

This report describes a case of metastatic clear-cell RCC presenting with severe hypercalcemia (serum calcium level >15 mg/dL), highlighting the diagnostic challenges inherent to such presentations. This case emphasizes the importance of early detection, comprehensive imaging, and a multidisciplinary treatment approach that integrates targeted therapies with supportive management to optimize clinical outcomes.

## Methods

This case report was prepared in accordance with the SCARE 2023 guidelines to ensure comprehensive reporting of clinical findings, diagnostic processes, therapeutic interventions, and outcomes^[7]^. The patient was managed at a tertiary academic medical center, reflecting a multidisciplinary approach involving oncology, radiology, and supportive care teams. Data were collected from electronic medical records, laboratory results, imaging studies, and histopathological reports. Informed consent for publication was obtained from the patient during the follow up period, in compliance with institutional ethical standards. All patient information has been de-identified to protect privacy.

## Case presentation

A 64-year-old Caucasian male with a medical history of hypertension, generalized anxiety disorder, exercise-induced asthma, thalassemia minor, and gastroesophageal reflux disease presented for a routine, well-adult examination at a tertiary academic medical center. He reported malaise, bloating, and unintentional weight loss of 3–4 pounds over the preceding 3–4 months. Notably, he had a history of excessive alcohol consumption (approximately 10 drinks per day for the past 4 years) and a family history of malignancies, including lung cancer in his father, skin cancer in his mother, and eye cancer in his aunt.

On initial physical examination, the patient was afebrile with a pulse of 75 bpm, respiratory rate of 16 breaths/min, and maintained oxygen saturation in room air at 96%. However, he was hypertensive with blood pressure readings of 194/96 mmHg (right arm) and 191/92 mmHg (left arm), and had mild pallor and dehydration. Outpatient laboratory evaluations revealed microcytic anemia on the complete blood count (CBC; see Table 1) and severe hypercalcemia with a serum calcium level of 15.1 mg/dL alongside an elevated creatinine of 1.81 mg/dL on the basic metabolic panel (BMP; see Table 1). Additional tests, including thyroid-stimulating hormone (TSH), HbA1c, and fasting lipid panel, were within normal limits.

**Table 1:** Initial blood investigations (CBC and BMP)

	Laboratory value	Reference range
**Complete blood count (CBC)**		
WBC count	7.9 × 10^3^/mcL	(3.4–10.8) × 10^3^/mcL
RBC count	5.02 × 10^6^/mcL	4.14–5.8 × 10^6^/mcL
Haemoglobin (Hb)	10.9 gm/dL	13.0–17.7 g/dL
Haematocrit (Hct)	38.1%	37.5–51.0%
RDW width	15.1%	11.6–15.4%
MCH	21.7 pg	26.6–33.0 pg
MCHC	28.6 gm/dL	31.5–35.7 gm/dL
MCV	76 fL	79–97 Fl
Platelet count	339 × 10^3^/mcL	(160–450) × 10^3^/mcL
**Basic metabolic panel (BMP)**		
Sodium	145 mmol/L	134–144 mmol/L
Potassium	4.2 mmol/L	3.5–5.2 mmol/L
Chloride	105 mmol/L	96–106 mmol/L
Carbon dioxide	24 mmol/L	20–29 mmol/L
Blood urea nitrogen (BUN)	22 mg/dL	8–27 mg/dL
Creatinine	1.81 mg/dL	0.76–1.27 mg/dL
BUN/Creatinine ratio	13	10–24
Calcium	15.1 mg/dL	8.6–10.2 mg/dL
Glucose	93 mg/dL	70–99 mg/dL
Alkaline phosphatase (ALP)	114 IU/L	44–121 IU/L
Bilirubin, total	0.8 mg/dL	0.0–1.2 mg/dL
Albumin	4.1 gm/dL	3.9–4.9 gm/dL
Globulin	2.3 gm/dL	1.5–4.5 gm/dL
Total protein	6.4 gm/dL	6.0–8.5 gm/dL
Albumin/globulin ratio	1.8	2.2
ALT	24 IU/L	0–44 IU/L
AST	20 IU/L	0–40 IU/L
Hemoglobin A1C (HbA1C)	4.5 %	4.5-5.6 %
TSH	3.4 mcIU/mL	0.450-4.500 mcIU/mL
Total cholesterol	150 mg/dL	100-199 mg/dL
Triglyceride	102 mg/dL	0-149 mg/dL
HDL cholesterol	41 mg/dL	>39 mg/dL
VLDL cholesterol	19 mg/dL	5-40 mg/dL
LDL cholesterol	90 mg/dL	0-99 mg/dL
**Parathyroid hormone profile**		
Parathyroid hormone (PTH)	4.2 pg/mL	9.0-73.0 pg/mL
Parathyroid hormone-related peptide (PTHrP) level	83 pg/mL	11-20 pg/mL

Abbreviations: WBC, white blood cell; RBC, red blood cell; MCV, mean corpuscular volume; MCH, mean corpuscular hemoglobin; RDW, red cell distribution width; mcL, microliter; MPV, mean platelet volume; L, liter; gm/dL, grams per deciliter; pg/cell, picograms per cell; fL, femtoliters; BUN, blood urea nitrogen; A/G ratio, albumin to globulin ratio; mcIU/mL, micro International Units per milliliter; mg/dL, milligrams per deciliter; gm/dL, grams per deciliter; mL/min/1.73 m^2^, milliliters per minute per 1.73 meters squared; mmol/L, millimoles per liter; IU/L, International Units per liter.

**Figure 1.**
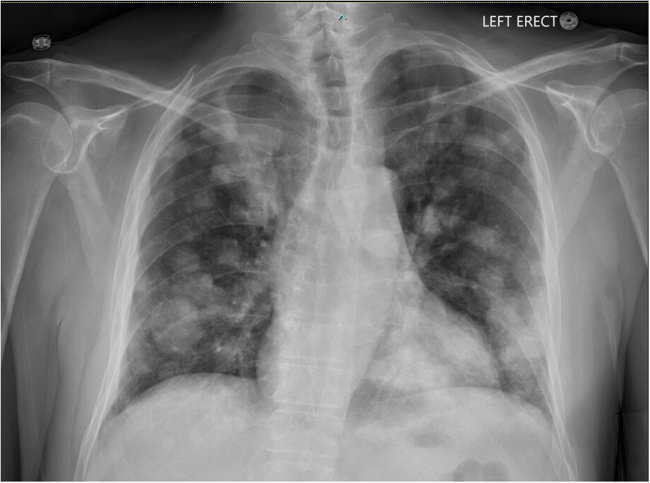
Chest X-ray with posteroanterior (PA) view demonstrating extensive bilateral nodular opacities concerning for metastatic disease.

The patient was urgently referred to the emergency department, where repeat serum calcium level was documented at 14.6 mg/dL. Further laboratory evaluation demonstrated a serum creatinine of 1.7 mg/dL, albumin of 3.8 g/dL, and hemoglobin of 10.9 g/dL (MCV: 76 fL). Initial management included aggressive hydration with 3 Liters of intravenous fluid (0.9% NS) boluses, followed by continuous infusion at 150 cc/h over the next two days. As part of the hypercalcemia workup, chest radiography (Fig. 1) revealed multiple bilateral nodular opacities associated with metastatic disease.

**Figure 2.**
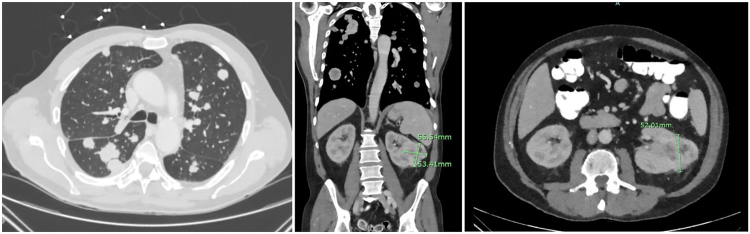
Contrast-enhanced CT of the Chest, Abdomen, and Pelvis. (A) CT imaging revealing multiple pulmonary nodules. (B) CT showing a lobulated, exophytic heterogeneous enhancing soft tissue mass in the left kidney measuring approximately 5.5 × 5.2 × 5.3 cm. (C) CT demonstrating similar renal mass findings with concerns for portal vein thrombus.

Subsequent contrast-enhanced computed tomography (CT) of the chest, abdomen, and pelvis (Fig. 2a-2c) identified a 5.5 × 5.0 × 5.3 cm lobulated mass in the left kidney, suggestive of renal cell carcinoma, with evidence of left renal vein thrombosis, multiple pulmonary nodules with central necrosis, mediastinal invasion by left upper lobe metastases, and an enhancing nodule within the right adrenal gland. Abdominal visceral duplex ultrasonography confirmed the presence of a left renal vein thrombus, presumed to be a tumor thrombus, thereby obviating the need for anticoagulation.

Further laboratory analysis demonstrated suppressed parathyroid hormone (PTH) levels (<4.2 pg/mL; reference range: 9.0–73.0 pg/mL) and an elevated parathyroid hormone-related peptide (PTHrP) level of 83 pg/mL (reference range: 11–20 pg/mL) (Table 1).

Following the clinical diagnosis, the patient was referred for comprehensive oncological evaluation. Histopathological analysis of a percutaneous, computed tomography (CT)-guided, three-core needle biopsy specimen obtained from a left upper lobe pulmonary lesion confirmed metastatic clear-cell renal cell carcinoma. Systemic oncotherapy was promptly initiated, comprising combination immunotherapy with pembrolizumab (200 mg intravenously every three weeks) and targeted tyrosine kinase inhibition with lenvatinib (20 mg orally daily), administered under the supervision of a multidisciplinary oncology team with extensive experience in managing advanced renal cell carcinoma. To address the presenting severe hypercalcemia, intravenous zoledronic acid (4 mg) was administered by the oncology team to achieve initial biochemical control. Concurrent iron deficiency anemia was managed with intravenous iron sucrose therapy, administered according to the hemoglobin deficit and iron indices. Furthermore, for pre-existing systemic hypertension, pharmacological management was initiated with amlodipine (10 mg orally once daily) and carvedilol (6.25 mg orally twice daily). This antihypertensive regimen resulted in a demonstrable improvement in blood pressure parameters and contributed to subsequent laboratory value normalization, as documented at the time of hospital discharge (Table 2).

**Table 2:** Discharge laboratory values

	Laboratory value	Reference range
Sodium	144 mmol/L	134–144 mmol/L
Potassium	3.8 mmol/L	3.5–5.2 mmol/L
Chloride	111 mmol/L	96–106 mmol/L
Carbon dioxide	24 mmol/L	20–29 mmol/L
Blood urea nitrogen (BUN)	12 mg/dL	8–27 mg/dL
Creatinine	1.60 mg/dL	0.76–1.27 mg/dL
BUN/Creatinine ratio	12	10–24
Calcium	13.5 mg/dL	8.6–10.2 mg/dL

**Figure 3.**
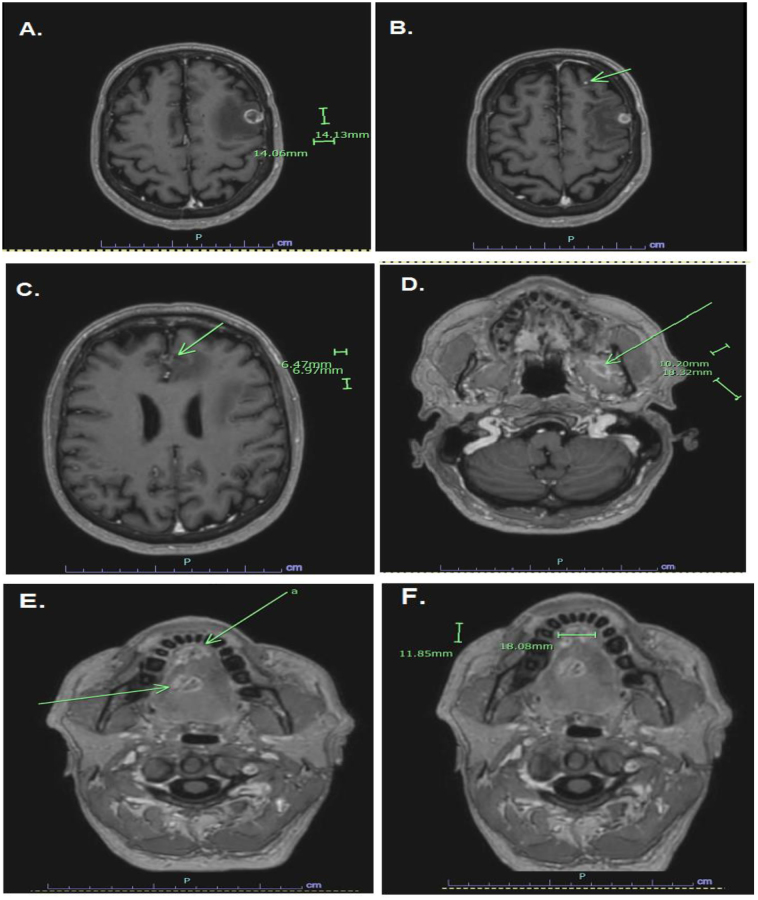
MRI of the brain. (A) 1.4 × 1.4 cm enhancing lesion in the left frontal lobe with surrounding vasogenic oedema; (B) 0.7 × 0.6 cm peripherally enhancing lesion in the left parasagittal frontal lobe with adjacent oedema and local mass effect; (C) Punctate focus of enhancement in the left parietal lobe; (D) 1.8 × 1.0 cm heterogeneously enhancing lesion in the left masticator space; (E) 1.5 × 1.8 × 1.7 cm enhancing lesion in the mid-tongue; (F) 1.2 × 1.8 cm enhancing lesion in the anterior tongue/floor of mouth.

**Figure 4.**
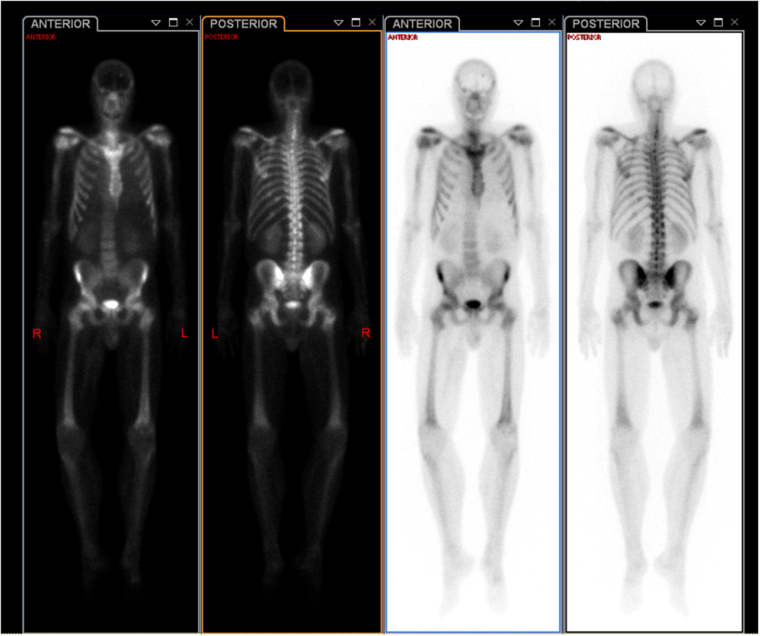
Whole-Body Bone Scan demonstrating abnormal MDP uptake in several posterolateral lower left ribs, indeterminate for metastasis versus chronic fractures, with no evidence of widespread osteoblastic metastases.

Additionally, MRI of the brain done for headache revealed multiple enhancing lesions with surrounding vasogenic edema in the left frontal and parietal lobes, mid-tongue, floor of mouth, and left masticator space (Fig. 3 A–F), while a whole-body bone scan demonstrated abnormal MDP uptake in the left posterolateral lower ribs, indeterminate for metastasis versus chronic fractures (Fig. 4).

Palliative radiation and dexamethasone were administered to the cerebral lesions with temporary suspension and subsequent resumption of targeted therapies. Follow-up imaging after 4 months demonstrated an interval reduction in the size of the primary renal mass, regression of pulmonary metastases, and improvement in cerebral edema, underscoring the benefit of a multidisciplinary treatment approach.

## Discussion

This case highlights the complexity of managing advanced renal cell carcinoma (RCC) when paraneoplastic hypercalcemia is present, a phenomenon reported in approximately 17% of RCC cases that is consistently associated with aggressive tumor behavior^[5,6]^. In our patient, markedly elevated serum calcium levels, predominantly driven by parathyroid hormone-related peptide (PTHrP) overproduction with concurrent suppression of parathyroid hormone (PTH), served as an early and ominous indicator of extensive disease burden. Unlike the classical presentation of RCC, typically characterized by hematuria, flank pain, or a palpable mass, the detection of hypercalcemia in our patient was the principal clinical clue, a finding that aligns with previous reports linking metabolic disturbances to poorer prognoses and advanced tumor biology^[5,8]^. This observation aligns with several studies that have documented poorer prognoses in RCC patients who present with metabolic disturbances^[9,10]^, highlighting the critical importance of early detection and prompt intervention.

Variability in the underlying mechanisms of hypercalcemia in RCC has been well documented^[6]^. While some reports suggest a classical humoral profile mediated by elevated PTHrP levels, other cases of hypercalcemia reveal alternative pathways, such as calcitriol-mediated hypercalcemia^[5]^. For instance, one case^[8]^ described a patient with non-suppressed PTH, low PTHrP, and elevated vitamin D metabolite levels, highlighting the need to consider multiple concurrent etiologies in the differential diagnosis. In contrast, our patient exhibited a pronounced PTHrP-mediated mechanism with marked suppression of PTH, which aligns more closely with the traditional humoral hypercalcemia of malignancies. This disparity highlights the heterogeneity in the pathophysiology of RCC-associated hypercalcemia and reinforces the importance of comprehensive diagnostic evaluation that includes measurements of PTH, PTHrP, and vitamin D metabolites.

There are notable differences in clinical outcomes and management strategies between our report and other published cases. One report^[5]^ described a patient with small, localized RCC and paraneoplastic hypercalcemic coma who achieved normalization of serum calcium following partial nephrectomy, while another case^[11]^ described a younger patient with metastatic RCC where cytokine-mediated effects such as those from IL-6 and TNF-α played a significant role in hypercalcemia. In contrast, our case was characterized by extensive metastatic involvement and a robust PTHrP-driven process, necessitating a multidisciplinary approach that included aggressive supportive care, systemic immunotherapy, and targeted tyrosine kinase inhibition. These differences in biochemical profiles, tumor burden, and treatment responses underscore the need for individualized management protocols and further prospective studies to optimize the therapeutic strategies for RCC-associated hypercalcemia.

Our diagnostic strategy, integrating advanced imaging modalities such as contrast-enhanced computed tomography and duplex ultrasonography with comprehensive laboratory evaluations of serum calcium, PTH, and PTHrP, followed by confirmatory histopathological analysis via percutaneous biopsy, mirrors contemporary best practices^[11,12]^ and underscores that a thorough, multimodal workup is imperative in cases of suspected paraneoplastic syndromes. This approach not only ensures accurate delineation of the primary renal lesion and its extent but also facilitates early identification of metastatic spread and vascular involvement^[10]^, which are critical for tailoring optimal therapeutic interventions. Recent studies have emphasized that the integration of biochemical and imaging data is essential to differentiate between various etiologies of hypercalcemia and guide subsequent management decisions^[13,14]^.

The aggressive management strategy employed in our patient further reinforced the importance of a coordinated multidisciplinary treatment paradigm. Prompt initiation of intravenous hydration, coupled with bisphosphonate therapy to counteract osteoclastic bone resorption, sets the foundation for stabilizing metabolic derangements^[15]^. The subsequent incorporation of modern systemic treatments, specifically, the immune checkpoint inhibitor pembrolizumab and the tyrosine kinase inhibitor lenvatinib, resulted in significant tumor regression and sustained control of hypercalcemia, outcomes that are consistent with reports in the literature^[16]^. Nevertheless, the optimal sequencing and duration of these therapeutic modalities remain under active investigation, highlighting the need for future prospective studies to refine the treatment protocols and improve prognostic models.

Our case reinforces the importance of early recognition and comprehensive evaluation of hypercalcemia as a harbor of aggressive RCC. The integration of clinical, biochemical, imaging, and histopathological data is vital for devising individualized treatment plans, which may ultimately alter the natural history of this formidable malignancy. Continued research, including well-designed prospective trials, is essential to further elucidate the molecular underpinnings of RCC-associated hypercalcemia and optimize therapeutic strategies for this high-risk patient population.

## Conclusion

Early recognition of paraneoplastic hypercalcemia in renal cell carcinoma is critical, as it often signifies advanced disease and necessitates prompt, aggressive intervention. A multidisciplinary approach combining targeted immunotherapy with supportive measures may improve outcomes and warrant further prospective investigations.

## Data Availability

Data sharing is not applicable to this article as no datasets were generated or analyzed during the current study.

## Patient Perspective

The patient expressed agreement with the proposed treatment plan, including aggressive hydration, zoledronic acid, and combination immunotherapy with pembrolizumab and lenvatinib. Following stabilization of his severe hypercalcemia, he reported feeling happy with the initial symptom relief and the multidisciplinary care provided. He consented to the use of his case for educational purposes, reflecting his satisfaction with the approach and desire to contribute to medical knowledge.
